# The relationship between antipsychotic medication adherence and patient outcomes among individuals diagnosed with bipolar disorder: a retrospective study

**DOI:** 10.1186/1744-859X-8-7

**Published:** 2009-02-18

**Authors:** Maureen J Lage, Mariam K Hassan

**Affiliations:** 1 Health Metrics Outcomes Research, Groton, CT, USA; 2 AstraZeneca Pharmaceuticals LP, Wilmington, DE, USA

## Abstract

**Background:**

Reducing hospitalizations and emergency room visits is important to improve patient outcomes. This observational study examined the association between adherence to antipsychotics and risk of hospitalizations and emergency room (ER) visits among patients with bipolar disorder.

**Methods:**

Claims data from commercial healthcare plans (Pharmetrics; January 2000 to December 2006) for patients with bipolar disorder receiving an antipsychotic prescription were examined. Adherence was analyzed over a 12-month follow-up period after the receipt of first prescription of an antipsychotic. Adherence to antipsychotics was measured by the medication possession ratio (MPR). The MPR was calculated as the number of days that an antipsychotic medication was filled as compared with the total number of days during the follow-up period. Logistic stepwise regressions examined the association between achievement of various adherence goals and patient outcomes (hospitalization or ER visit for mental health or any reason).

**Results:**

In total, 7,769 patients with bipolar disorder were included. The mean MPR was 0.417, with 61.7% of individuals having an MPR < 0.50, and 78.7% an MPR < 0.75. As adherence improved, the risk of hospitalization or ER visit declined. A significant reduction in the risk of hospitalization (odds ratio (OR) 0.85, 95% confidence interval (CI) 0.75 to 0.98) or an ER visit (OR 0.84, 95% CI 0.74 to 0.96) for any cause was associated with an MPR ≥ 0.75. An MPR ≥ 0.80 was associated with a significant reduction in the risk of a mental health-related hospitalization (OR 0.82, 95% CI 0.70 to 0.95), while an MPR ≥ 0.90 was associated with a significant reduction in risk of a mental health-related ER visit (OR 0.71, 95% CI 0.54 to 0.91).

**Conclusion:**

Patients with lower antipsychotic adherence were at greater risk of hospitalizations and ER visits. Thus, any efforts to increase adherence, even in small increments, can be helpful in decreasing these risks.

## Introduction

As the sixth leading cause of disability worldwide [1], bipolar disorder affects approximately 5.7 million American adults, or about 2.6% of the US population aged 18 and older, annually [2]. A recent study of records from the annual National Hospital Discharge Survey (NHDS) reported that the population-adjusted rates of hospital discharges with a primary diagnosis of bipolar disorder grew significantly between 1996 and 2004 among all age groups [3], with the adult rate rising markedly, by 56%. While bipolar disorder has a spectrum of expression, the classic form of the illness, in which a person experiences recurrent episodes of mania and depression, is bipolar I disorder [4].

The direct costs of bipolar disorder are substantial and include inpatient hospitalization, outpatient general and specialist visits, nursing home, intermediate, and domiciliary care, medication, substance abuse treatment, and costs of supported living [5]. To date, two US cost-of-illness studies, one prevalence-based and the other incidence-based, have been conducted specifically on bipolar disorder [5,6]. The prevalence-based study estimated the direct, annual costs of the disease to be $7.6 billion (in 1991 US dollars) [5], while the incidence-based study reported the lifetime, direct costs for cases of bipolar disorder diagnosed in 1998 to be $13 billion [6]. When considering the costs of medical care, a study that assessed health care claims from a database of 1.66 million people insured through more than 900 employers determined bipolar disorder to be the most expensive behavioral health diagnosis [7]. Moreover, it has been shown to be the most expensive mental health condition among employees of six large US corporations [8]. In a recent analysis, direct, per-patient costs were $3,000 (in 2004 US dollars) higher for patients with bipolar disorder than for patients with non-bipolar depression (p < 0.001), with the primary differences observed for psychiatric medication ($1,641 vs $507) and psychiatric hospitalization ($1,187 vs $241) [9]. Other research has also shown that inpatient care is a key driver of medical costs for patients with mental illness and, in particular, bipolar disorder [5,10].

As the costs of bipolar disorder are very high, both to the patient and the health care community, finding ways to decrease unnecessary financial and personal costs is important. Though, bipolar disorder poses significant treatment challenges due to the severity and varied nature of the illness, patients can be stabilized and managed with proper treatment [11,12]. In most cases, bipolar disorder can be better controlled and outcomes are improved if the patients are adherent to their medication regimen [13-16]. However, medication adherence is a critical problem among bipolar disorder patients. Adherence to mood stabilizers and valproates, commonly used pharmacotherapy in bipolar disorder, is about 20 to 60% [13,15,17,18]. Adherence to atypical antipsychotics, recently approved for bipolar disorders, is relatively less studied; although one study did report that nearly half (48.1%) of patients taking antipsychotics to treat bipolar disorder are partially adherent or non-adherent with their medications [19].

The objective of our study is to evaluate various levels of adherence to antipsychotics among bipolar disorder patients and examine if increasing antipsychotic adherence can help to diminish the risk of hospitalization or emergency room (ER) visits. In this study, we examined the impact of different degrees of antipsychotic medication adherence on the risk of hospitalization or ER visits among individuals diagnosed with bipolar disorder.

## Methods

### Data collection and study population

This retrospective cohort study evaluated claims data from the Pharmetrics database (Watertown, MA, USA) covering the period 1 January 2000 to 31 December 2006. The fully de-identified and Health Insurance Portability and Accountability Act (HIPAA) compliant database contains information on patient demographics and hospitalizations, outpatient service utilization, and outpatient pharmacy data from over 75 different managed care organizations and more than 55 million individuals.

Data were obtained from patients aged between 18 and 64 years with bipolar disorder (identified by paid claims with the International Classification of Diseases, Ninth Revision, Clinical Modification (ICD-9-CM), codes 296.4× to 296.8×) who had received an antipsychotic prescription. The first date of a paid claim for an antipsychotic was defined as the index prescription. All patients included in the study were required to have at least 6 months of continuous enrollment in the same health care plan prior to and 12 months after the date of the index prescription. Given the duration of the preindex and postindex periods, as well as the data collection period, the index date was required to be between 1 July 2000 and 1 January 2006.

Patients with a diagnosis of dementia (ICD-9-CM, code 290.xx) or schizophrenia (ICD-9-CM, code 295.xx) were excluded from the analysis in order to reduce the probability of including patients who were misdiagnosed.

### Measures of adherence and outcomes

The medication possession ratio (MPR) was utilized as a measure of adherence, and was calculated as the number of unique days an antipsychotic medication was prescribed in the postindex period divided by the number of days in the same period [20-22]. Therefore, an MPR of 1 indicates that the patient was prescribed an antipsychotic over the complete 12 months following initiation, and that these prescriptions were filled 100% of the time.

The main outcome measures in this analysis were the probability of hospitalization or ER visit for any cause. Additional outcome measures examined the probability of hospitalization or an ER visit with an accompanying mental health diagnosis (ICD-9-CM, codes 290.xx to 319.xx).

### Statistical analysis

A series of stepwise logistic multivariate analyses were conducted to assess the relationship between patient outcomes and progressive increases in MPRs (categorized into adherence thresholds of 0.25, 0.50, 0.70, 0.75, 0.80, 0.90, and 0.95) with adjustment for the effect of a wide range of confounding factors. Thus, the analysis controlled for patient demographic characteristics; type of bipolar disorder; patient general health status (including Charlson Comorbidity Index score [23,24], total number of diagnoses, and total number of outpatient prescription medications received in the postindex period); psychiatric prescriptions and specific comorbidities diagnosed in the preindex period (panic disorder, obsessive compulsive disorder and generalized anxiety disorder, attention-deficit hyperactivity, depression, substance abuse, obesity, cardiovascular disease, diabetes, hypertension, and high cholesterol).

MPR and all other variables that reached a threshold of 90% significance were included in the stepwise logistic regressions. By estimating a series of regressions with various MPR thresholds, the multivariate analyses allowed for an examination of how changes in the MPR affect patient outcomes, without artificially compelling a linear relationship between MPR and outcomes, or arbitrarily determining that any particular MPR threshold is appropriate. All analyses were conducted using SAS, version 9.1 (SAS, Cary, NC, USA). Statistical significance was accepted at p ≤ 0.05.

## Results

### Patient characteristics

Table 1 presents the demographic and clinical characteristics of the 7,769 patients with bipolar disorder included in the study.

**Table 1:** Demographic and clinical characteristics of patients with bipolar disorder (n = 7,769)

**Variable**		
	Mean	SD
Patient characteristics		
Age, years	39.71	12.74
General health, preindex period:		
Charlson Comorbidity Index score	0.38	0.99
Diagnoses, n	8.20	6.19
Prescriptions, n	6.40	5.38
	n	%
Sex:		
Female	4,985	64.17
Male	2,784	35.83
Region:		
Midwest	2,161	27.82
Northeast	2,299	29.59
South	2,262	29.12
West	1,047	13.48
Insurance type:		
Commercial	7,334	94.40
Other	435	5.60
Bipolar disorder type:		
Depressed	1,362	17.53
Manic	923	11.88
Mixed	1,424	18.33
Other	4,060	52.26
General health, preindex period:		
Hospitalized	2,165	27.87
Comorbidities, preindex period:		
Panic disorder	356	4.58
Obsessive compulsive disorder	218	2.81
Generalized anxiety disorder	584	7.52
Substance abuse	1,625	20.92
Obesity	268	3.45
Depression	2,775	35.72
Cardiovascular disease	198	2.55
Diabetes	452	5.82
Hypertension	1,101	14.17
High cholesterol	425	5.47
Attention-deficit/hyperactivity	390	5.02
Compliance:		
MPR ≥ 0.25	4,540	58.44
MPR ≥ 0.50	2,776	35.73
MPR ≥ 0.75	1,509	19.42
MPR ≥ 0.80	1,229	15.82
MPR ≥ 0.90	651	8.38
MPR ≥ 0.95	333	4.29

MPR, medication possession ratio.

The mean MPR for this cohort was 0.417 (41.7%), with 61.9% having an MPR ≤ 0.50 and 78.7% having an MPR ≤ 0.75. Among the patients included in this cohort, the mean age was 40 years; 64% were female, and the majority were commercially insured (94.4%), with a diagnosis of bipolar type other (52.3%). An examination of the general health status of this population in the 6 months prior to antipsychotic medication initiation revealed that 27.9% had been hospitalized, and that patients had received a mean of 6.2 distinct diagnoses and 5.4 outpatient prescriptions. Depression (35.7%), substance abuse (20.9%), and hypertension (14.2%) were the most common comorbid conditions among patients in the 6 months before antipsychotic medication initiation.

An evaluation of antipsychotic medication use in the 12 months following initiation of antipsychotic treatment showed that the vast majority of individuals were prescribed atypical antipsychotics (Table 2). Notably, 95% of patients were prescribed at least one atypical antipsychotic in the postindex period, while only 10% were prescribed a typical antipsychotic over the same time period. Of the atypical antipsychotic medications used, patients were most likely to be prescribed quetiapine (43.5%), olanzapine (32.2%), or risperidone (26.8%). Of the typical antipsychotic medications, prochlorperazine (6.7%) and haloperidol (1.5%) were the most frequently prescribed medications.

**Table 2:** Antipsychotic medication use among patients with bipolar disorder

	**Use of drug**		**Days prescribed**	
	
**Medication**	**n**	**%**	**Mean**	**SD**
Atypicals:				
Aripiprazole	1,354	17.43	125.71	120.88
Clozapine	6	0.08	140.67	176.62
Olanzapine	2,502	32.20	120.76	121.26
Fluoxetine/Olanzapine	316	4.07	114.78	139.2
Quetiapine	3,376	43.45	156.85	141.55
Risperidone	2,084	26.82	124.04	124.49
Ziprasidone	532	6.85	117.93	115.47
Any atypical	7,378	94.97	175.08	158.77
Typicals:				
Chlorpromazine	37	0.48	99.68	134.54
Droperidol	1	0.01	1	
Fluphenazine	12	0.15	87.25	84.06
Haloperidol	114	1.47	82.68	101.78
Loxapine	13	0.17	188.46	192.04
Mesoridazine	0	0		
Molindone	1	0.01	60	
Perphenazine	63	0.81	127.24	128.42
Pimozide	1	0.01	30	
Piperacetazine	0	0		
Prochlorperazine	518	6.67	14.96	30.975
Promazine	0	0		
Thioridazine	16	0.21	79	73.50
Thiothixene	28	0.36	162.07	164.60
Trifluoperazine	10	0.13	268.5	226.81
Triflupromazine	0	0		
Any typical	779	10.03	5.27	35.01

### Adherence (medication possession ratio) and hospitalization risk

**Figure 1:**
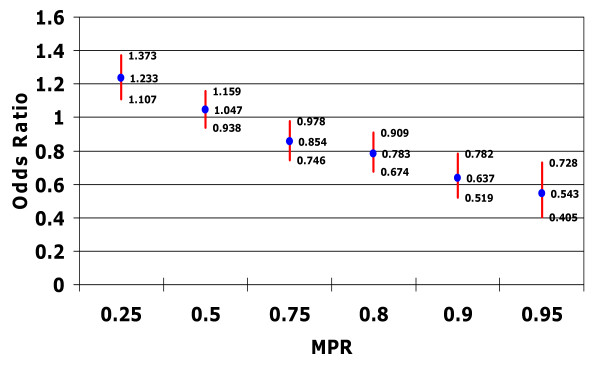
**Relationship between medication possession ratio and risk of hospitalization for any cause**. Controlling for confounding factors such as patient demographic characteristics; type of bipolar disorder; patient general health status (including Charlson Comorbidity Index score, total number of diagnoses, and total number of outpatient prescription medications received in the postindex period) psychiatric prescriptions; and specific comorbidities diagnosed in the preindex period.

**Figure 2:**
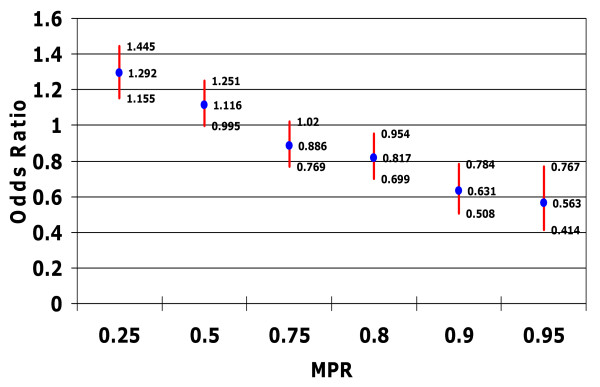
**Relationship between medication possession ratio and risk of hospitalization with mental health diagnosis**. Controlling for confounding factors such as patient demographic characteristics; type of bipolar disorder; patient general health status (including Charlson Comorbidity Index score, total number of diagnoses, and total number of outpatient prescription medications received in the postindex period) psychiatric prescriptions; and specific comorbidities diagnosed in the preindex period.

The associations between patient adherence, as measured by MPR, and the probability of hospitalization for any cause or with an accompanying mental health diagnosis are presented in Figures 1 and 2.

Patients with an MPR threshold of 0.25 had significantly higher odds of hospitalization for any cause compared with patients with an MPR < 0.25 (odds ratio (OR) 1.23, 95% confidence interval (CI) 1.11 to 1.37) as well as significantly higher odds of a mental health-related hospitalization (OR 1.29, 95% CI 1.16 to 1.45). In contrast, patients who reached an MPR threshold of 0.75 had significantly lower odds of hospitalization for any cause (OR 0.85, 95% CI 0.75 to 0.91), and those who reached an MPR threshold of at least 0.80 demonstrated a significant reduction in the odds of a mental health-related hospitalization (OR 0.82, 95% CI 0.70 to 0.95).

Thus, as patients achieved a higher MPR threshold, the risk of hospitalization declined (Figures 1 and 2). Patients who achieved an MPR threshold of at least 0.75 had an approximate 15% reduction in the odds of being hospitalized (p < 0.05), while those who achieved an MPR threshold of 0.90 or 0.95 had a 36% (p < 0.05) or 46% (p < 0.05) reduction in the odds of hospitalization, respectively.

### Adherence (medication possession ratio) and risk of emergency room visits

**Figure 3:**
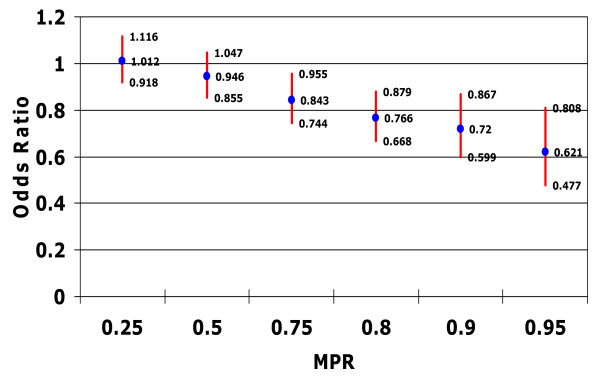
**Relationship between medication possession ratio and risk of emergency room visit for any cause**. Controlling for confounding factors such as patient demographic characteristics; type of bipolar disorder; patient general health status (including Charlson Comorbidity Index score, total number of diagnoses, and total number of outpatient prescription medications received in the postindex period) psychiatric prescriptions; and specific comorbidities diagnosed in the preindex period.

Figure 3 illustrates the association between patient adherence and the odds of an ER visit for any cause. At MPR thresholds of 0.25 or 0.50, the relationship between patient medication adherence and lower odds of ER visits for any cause did not reach significance. However, an MPR threshold of at least 0.75 was associated with significant reductions in the odds of an ER visit for any cause (OR 0.84, 95% CI 0.74 to 0.96). Thus, higher adherence thresholds (MPR > 0.75) resulted in a reduction in the risk of an ER visit for any cause, with an MPR of at least 0.75 associated with a 16% lower risk of visiting the ER (p < 0.05) (Figure 3). Moreover, patients with an MPR of at least 0.95 had 38% lower odds of visiting the ER (p < 0.05).

**Figure 4:**
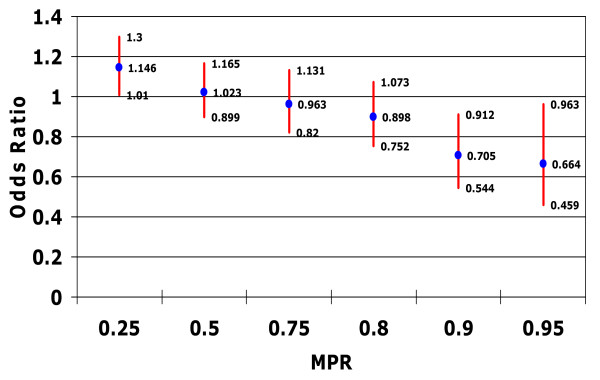
**Relationship between medication possession ratio and risk of emergency room visit with mental health diagnosis**. Controlling for confounding factors such as patient demographic characteristics; type of bipolar disorder; patient general health status (including Charlson Comorbidity Index score, total number of diagnoses, and total number of outpatient prescription medications received in the postindex period) psychiatric prescriptions; and specific comorbidities diagnosed in the preindex period.

Evaluation of the association between medication adherence and ER visits with an accompanying mental health-related diagnosis revealed that, as MPR thresholds increase, the odds of a mental health-related ER visit reduced (Figure 4). Contrary to the results for ER visits for any cause, a significant reduction in the odds of an ER visit for mental health reasons was not observed until patients reached a threshold of at least 0.90 (OR 0.71, 95% CI 0.54 to 0.91).

As a test of the robustness of the results reported here, the relationship between medication adherence, and the risk of hospitalization or an ER visit with an accompanying diagnosis of bipolar disorder was also examined. The observed results were generally consistent with the results from the analyses of the risk of mental health-related hospitalizations or ER visits (data not shown).

## Discussion

In this retrospective claims-based study, more than half of the 7,769 patients with bipolar disorder took their antipsychotic medication less than half of the time (61.9% had an MPR of less than 0.50), with the vast majority (78.7%) taking their medication less than 75% of the time. These observations are consistent with previous research indicating low levels of adherence to antipsychotics [19] and mood stabilizers [25] among patients with bipolar disorder. Alongside these findings, higher levels of adherence to antipsychotic medication were found to be associated with better patient outcomes, both in terms of hospitalizations and visits to the ER.

An examination of hospitalization outcomes revealed that, as patients achieved a higher MPR threshold, the odds of hospitalization for any cause as well as mental health-related hospitalizations, decreased. For instance, patients who achieved an MPR threshold of at least 0.75 had an approximate 15% reduction in the odds of being hospitalized for any cause (p < 0.05), while those who achieved an MPR threshold of 0.90 or 0.95 had a 36% or 46% (both p < 0.05) reduction in the odds of hospitalization, respectively.

While no previous study of individuals with bipolar disorder has reported on the relationship between adherence to antipsychotic medication and patient outcomes, previous research among patients with schizophrenia has shown partial or non-adherence to antipsychotic medication to be associated with higher rates of hospitalization [26]. In addition, several studies among patients with bipolar disorder have found a link between non-adherence to prescribed medication and hospitalization. An analysis of factors leading to hospitalization among elderly patients with bipolar mania found lack of adherence with prescribed psychiatric medication (for example, mood stabilizers) to be a major factor [14]. A study of adherence and outcomes among patients with bipolar disorder who were receiving antipsychotics, lithium, and antidepressants reported hospitalization rates of 73% for those classified as irregular medication users compared with 31% for regular users [15]. Similarly, an examination of adherence to mood stabilizers among individuals with mood disorders found hospital admission rates of non-adherent patients to be 81.2%, compared with a rate of only 9.7% among adherent individuals [16], while another study found non-adherence to mood-stabilizing medication to be a cause of relapse among patients with bipolar disorder [27]. Although these earlier studies did not primarily focus upon antipsychotic medications, the consistency of the findings indicates the importance of compliance to any treatment protocol.

Patients with an MPR of at least 0.75 had 16% lower odds of visiting the ER (p < 0.05), while those with an MPR of at least 95% had 38% lower odds of visiting the ER (p < 0.05). As observed with ER visits for any cause, an examination of the association between medication adherence and ER visits with an accompanying mental health-related diagnosis revealed that, as MPR thresholds increase, the odds of an ER visit decline. However, unlike the results for ER visits for any cause, a significant reduction in the odds of an ER visit for mental health reasons was not achieved until patients reached a threshold of at least 0.90 (OR 0.71, 95% CI 0.54 to 0.91). In comparison, an earlier study of the relationship between adherence to traditional mood-stabilizing therapy (lithium, valproate, carbamazepine, lamotrigine, oxcarbazepine) and health care utilization among patients with bipolar disorder, found adherence below 80% to be associated with a significantly greater risk of mental health-related ER visits (OR 1.98, 95% CI 1.38 to 2.84) [28]. This difference in results may indicate that the adherence threshold is higher for antipsychotic medications than for traditional mood-stabilizing therapy, although further research is needed before reaching a definitive conclusion.

One advantage of this study is that it allowed for an examination of effects on patient outcomes with various adherence thresholds. This study is in contrast to previous studies that defined adherence based upon a specific MPR threshold without necessarily explaining the choice of such a threshold [29-31]. Furthermore, it has been argued that 'the use of arbitrary categories of good and poor compliance (often set at 80%) usually was unsupported by research documenting the appropriateness of the cutoff for a specific medication class or disease' [32].

The findings presented here should be interpreted within the context of the limitations of the study design. This analysis was conducted using an administrative claims database, and included only patients with medical and outpatient prescription benefit coverage. The results, therefore, may not generalize well to other populations. Additionally, the use of diagnostic codes may be less rigorous than formal diagnostic assessments for identifying patients. Although analyses were adjusted for differences in bipolar disorder type, general health, and comorbidities, it was not possible to control for disease severity. The utilization of medical claims data precluded the inclusion of patient assessments and thus outcome measures related to quality of life, caregiver burden, or any of the other indirect costs associated with bipolar disorder were not included in this study. This investigation examined adherence to antipsychotic medications alone and did not account for prescribed changes in treatment protocol. Therefore, patients switched by their physicians from an antipsychotic to a different type of drug during the study period would have been viewed as non-adherent, even if they were fully compliant with their prescribed therapy. Finally, this study focused on both atypical and conventional antipsychotics, without controlling for class or exact type of medication. However, the results were largely driven by atypical antipsychotic medications, as demonstrated by 95% of the patient population receiving this class of therapy.

## Conclusion

In summary, the results of this analysis indicate that, among patients with bipolar disorder, greater levels of adherence to therapy with antipsychotic medications are associated with better patient outcomes. Specifically, higher adherence thresholds were associated with lower chances of hospitalization and ER events. In view of the current evidence of poor adherence to long-term medication therapy [33-36], the findings of this study are encouraging as they show that the efforts to improve adherence, even in smaller increments, may improve patient outcomes.

## Competing interests

MH is employed by AstraZeneca and ML received financial compensation from AstraZeneca for this project.

## Authors' contributions

MH made substantial contributions to the conception and design of the study, acquisition of the data, interpretation of the data, and drafting of the manuscript. MJL made substantial contributions to the analysis of the data, interpretation of the data and drafting of the manuscript.
